# Treating Acne Scars in Fitzpatrick Skin Types I–VI Using a Novel 1550 nm Non‐Ablative Resurfacing Laser With Focal Point Technology

**DOI:** 10.1111/jocd.70620

**Published:** 2025-12-22

**Authors:** Jordan V. Wang, Hyemin Pomerantz, Thanh‐Nga Tran, Valerie Callender, Robert Weiss, Margaret Weiss, Leyda Bowes, Roy G. Geronemus

**Affiliations:** ^1^ Laser & Skin Surgery Center of Pennsylvania Devon Pennsylvania USA; ^2^ Office of Roy G Geronemus New York New York USA; ^3^ VivaSkin Dermatology and Aesthetics Wellesley Massachusetts USA; ^4^ Department of Dermatology, Wellman Laboratories of Photomedicine Harvard Medical School Boston Massachusetts USA; ^5^ Callender Dermatology and Cosmetic Center Glenn Dale Maryland USA; ^6^ Howard University College of Medicine Washington DC USA; ^7^ Maryland Dermatology Laser Skin and Vein Institute Hunt Valley Maryland USA; ^8^ Department of Dermatology University of Maryland College Park Maryland USA; ^9^ AVAVA, Inc Waltham Massachusetts USA

**Keywords:** acne scars, aesthetics, dermatology, laser resurfacing, rejuvenation

## Abstract

**Background:**

Acne scars have traditionally been treated with non‐ablative fractional resurfacing lasers. Traditional 1550 nm lasers can be limited in energy delivery, skin cooling, and safety across all skin types. A new‐generation 1550 nm non‐ablative resurfacing laser was recently developed, employing conical beams for high energy delivery through Focal Point Technology.

**Objective:**

To evaluate the utility of a 1550 nm non‐ablative resurfacing laser using Focal Point Technology to improve acne scars in Fitzpatrick skin types I–VI.

**Methods and Materials:**

A prospective, multi‐center clinical study investigated this novel resurfacing laser using 1–6 treatments.

**Results:**

Forty‐seven subjects with Fitzpatrick skin types I–VI were enrolled and treated. Mean age was 40.2 years, and 59.6% were women. Based on GAIS, 78.0% were rated as improved, with none worsening. Two of three blinded reviewers correctly identified photographs for 92.7% of subjects. Significant improvement in blinded ECCA score was observed (85.6 vs. 55.3; *p* < 0.0001), with 90.2% achieving at least a 15‐point improvement. Treatments were well tolerated.

**Conclusion:**

This new‐generation 1550 nm non‐ablative laser using Focal Point Technology with high energy delivery is safe and effective for acne scar treatment in all Fitzpatrick skin types. Its conical beams and cooling system significantly reduce hyperpigmentation risk in darker skin types.

## 1 Introduction

Acne represents an increasingly prevalent skin condition, especially in teenagers and young adults [1]. Acne breakouts and flares can often persist into later adulthood. Various medical interventions have been utilized over the years to manage this condition, including topical antibiotics, topical anti‐inflammatories, oral antibiotics, hormonal therapies, topical retinoids, and oral isotretinoin [2, 3, 4, 5, 6]. Laser interventions have also been recently added to our available options [7, 8, 9, 10, 11]. Unfortunately, many cases of acne can lead to significant scarring, which is especially the case when severe enough or if medical treatments are either ignored or delayed [12]. Acne scars have been associated with significant social and emotional distress as well as negative mental well‐being [13].

Acne scarring disproportionately impacts skin‐of‐color populations, presenting unique therapeutic challenges, including higher incidences of post‐inflammatory hyperpigmentation (PIH) and keloid formation [14, 15]. Up to 90% of acne patients with darker skin types experience PIH, which underscores the critical need for effective and safe treatments designed specifically to address these concerns [16].

Acne scars are generally categorized as hypertrophic, atrophic, and keloidal, with further breakdown of atrophic scars into rolling, boxcar, and icepick types. These are associated with disruption to collagen and elastin, extracellular matrix composition, and dermal architecture from upregulated inflammatory pathways [17]. Traditional clinical treatments include chemical peels, microneedling, needle subcision, resurfacing lasers, radiofrequency devices, and ultrasound devices [15, 18, 19]. The guiding principle of these treatments typically revolves around the stimulation of new collagen and elastin formation with subsequent dermal remodeling.

**FIGURE 1:**
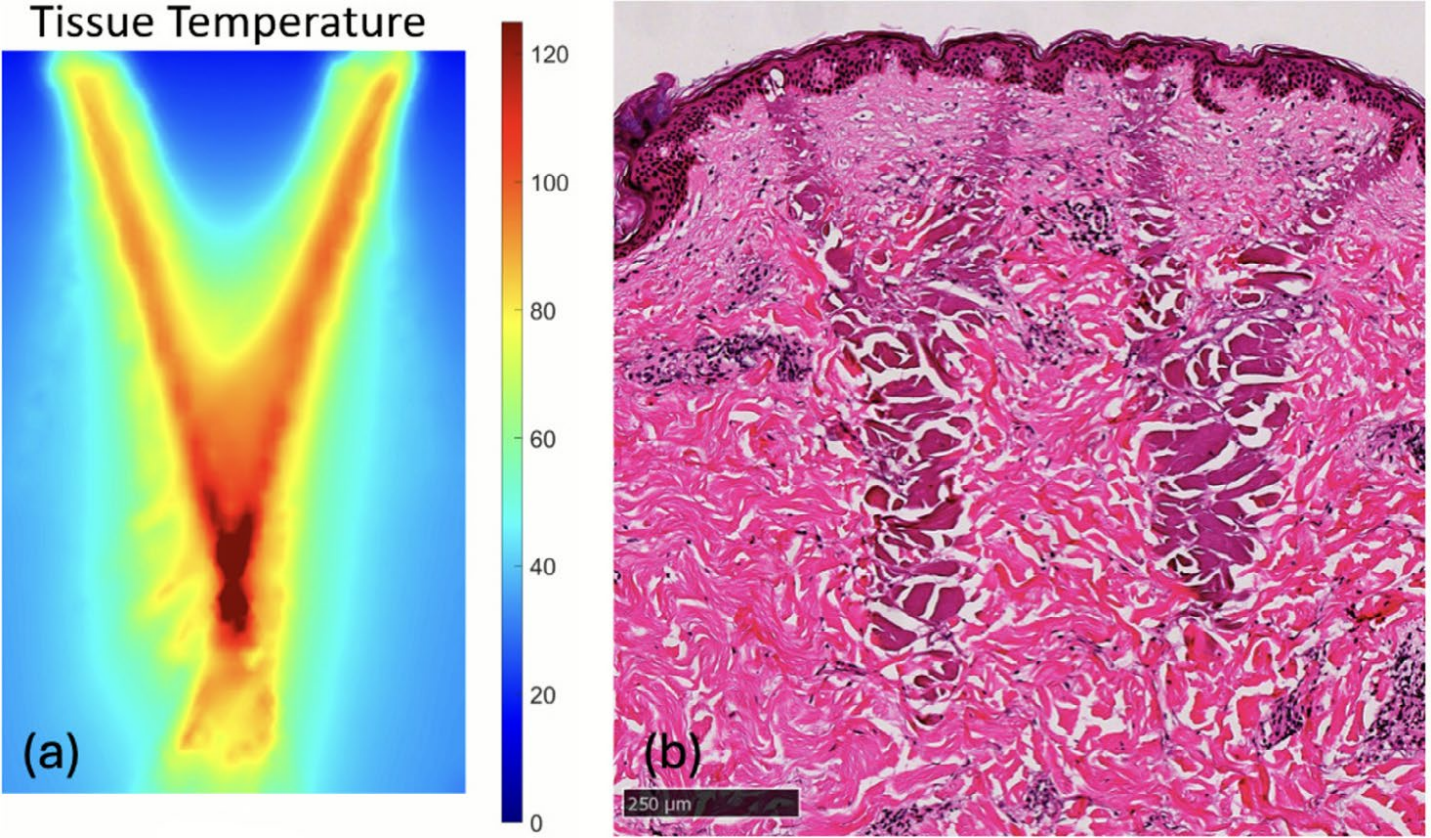
Delivery of high‐energy beams using focal point technology from a 1550 nm non‐ablative resurfacing laser (AVAVA, Waltham, MA) as demonstrated through (a) thermal mapping and (b) histology.

More recently, a novel 1550 nm non‐ablative resurfacing laser was developed, which employs conical beams for energy delivery through Focal Point Technology [20]. Due to the concentration of energy at the targeted depth and the reduced energy density at the superficial skin layers, higher energies (up to 150 mJ) can now be safely delivered through the skin compared to traditional 1550 nm non‐ablative resurfacing laser technology (up to 70 mJ) (Figure 1). This prospective, multi‐center, clinical study investigates the safety and efficacy of a new‐generation 1550 nm non‐ablative resurfacing laser that uses Focal Point Technology in treating acne scars. The unique delivery mechanism can allow for high‐energy treatments deep in the dermis to better target the pathophysiology of acne scars. With the inclusion of all skin types, this study can further evaluate the safety of these high‐energy treatments to mimic real‐world clinical practice.

## 2 Materials and Methods

Healthy subjects who were 18–72 years of age and seeking treatment for acne scars on the face were enrolled at three clinical sites, which included AVAVA clinic (Waltham, MA), Laser & Skin Surgery Center of New York (New York, NY), and Enchantress Dermatology (Miami, FL). This study was approved by an independent IRB. Informed consent was obtained from all subjects.

Subjects were excluded if they had hypersensitivity or seizure disorder to light; isotretinoin use within 6 months; topical retinoid use within 30 days; active acne, rosacea, or skin infection in the treatment area; significant inflammatory skin disease; history of herpes simplex in the treatment area; history of gold therapy; facial cosmetic procedures in the treatment area within 6 months; significant uncontrolled medical illness; history of immunosuppression or immunodeficiency; planned weight loss greater than five pounds; bleeding or coagulation disorder or taking medications affecting such; history of wound healing issues or keloid formation; history of skin cancer in the treatment area; current enrollment in another clinical trial; and if they were pregnant or planning to become pregnant, having given birth < 3 months ago, and/or breastfeeding. Intentional enrollment across Fitzpatrick types I–VI ensured a comprehensive evaluation of treatment outcomes and safety across diverse patient demographics, addressing previous research gaps where skin‐of‐color populations have historically been underrepresented [16, 21].

Each subject was to receive 1–6 treatments to the face, up to investigator discretion. Scheduled treatment intervals were 3–6 weeks, and follow‐up visits occurred at 1 and 3 months following the final treatment. Treatments were performed with this new‐generation 1550 nm non‐ablative resurfacing laser that uses Focal Point Technology (AVAVA, Waltham, MA). Prior to treatment, topical anesthetic was applied to the treatment area for up to 60 min. The face was then cleansed thoroughly with gentle facewash and 70% isopropyl alcohol. A thin coating of a bland hydrogel was applied to the face to allow for coupling of the laser energy, seamless gliding of the handpiece, and even distribution of cooling to protect the epidermis. The hydrogel was wiped off immediately after treatment.

Digital photographs were standardized with respect to subject positioning and ambient lighting. Photographs were taken at baseline prior to treatment, at all treatment visits, and at 1 and 3 months following the final treatment. Immediately following treatment, subjects were asked to rank their pain level using Visual Analog Scale (VAS) (10‐point scale).

Clinical improvement was rated by investigators using a Global Aesthetic Improvement Scale (GAIS) (5‐point scale; 1: much improved, 2: improved, 3: no change, 4: worse, 5: much worse). Three blinded, independent physicians were asked to review randomized photographs of subjects at baseline and at follow‐up. Blinded reviewers were instructed to correctly select pre‐ and post‐treatment photographs, and their inter‐rater agreement was measured. Two independent dermatologists were also asked to grade randomized photographs of subjects at baseline and at follow‐up using ECCA (échelle d'évaluation clinique des cicatrices d'acné) acne scar scale. Descriptive and statistical analyses were conducted, and top‐box scoring was used when appropriate.

## 3 Results

### 3.1 Subject Demographics

A total of 47 subjects were enrolled and underwent treatment. Mean age was 40.2 years (R: 21–72 years). Of all subjects, 59.6% were women. In terms of Fitzpatrick skin type (FST), 2.1% (*n* = 1) were Type I, 19.2% (*n* = 9) were Type II, 46.8% (*n* = 22) were Type III, 19.2% (*n* = 9) were Type IV, 8.5% (*n* = 4) were Type V, and 4.3% (*n* = 2) were Type VI.

### 3.2 Treatment Parameters

Subjects received 1–6 treatments (mean: 3.3 treatments). Up to two layers of the skin were targeted, including a deeper layer with zones of coagulation ranging 700–1300 μm in depth with significant epidermal sparing and a superficial layer with zones of coagulation ranging 200–400 μm in depth with less epidermal sparing.

For treatment parameters, the deeper layer had pulse energies of 50–150 mJ, while the superficial layer had pulse energies of 20–30 mJ. Early in the study, several treatments included only a single layer. For these treatments, the deep layer had a mean total energy of 4.050 kJ and the superficial layer had a mean total energy of 2.578 kJ. When both layers were treated at the same time later in the study, mean total energy delivered for the deeper and superficial layers was 3.867 kJ and 1.952 kJ, respectively, for a combined total of 5.819 kJ.

### 3.3 Investigator Clinical Rating

For GAIS clinical rating by investigators, the majority (78.0%) of all subjects were rated to be either Much Improved (1 out of 5) (41.5%) or Improved (2 out of 5) (36.6%). The remaining subjects (22.0%) were rated to have No Change (3 out of 5), with no subjects showing clinical worsening. The No Change ratings were primarily among initial study participants who were treated using lower energy settings and/or a single treatment pass during early protocol development.

### 3.4 Blinded Reviewer Rating

For photographic evaluation by three blinded, independent physician reviewers, 2 out of 3 were in agreement in correctly identifying the pre‐ and post‐treatment photographs for 92.7% of subjects, who were considered responders. All three reviewers were in agreement for 82.9% of subjects.

For photographic evaluation by two blinded, independent dermatologist reviewers, there was significant improvement in ECCA score of 30.3 points (85.6 points vs. 55.3 points; *p* < 0.0001). The vast majority (90.2%) had at least a 15‐point improvement and were considered responders.

### 3.5 Subject Pain Assessment

Pain management included only the use of topical anesthesia for up to 60 minutes. The reported overall mean pain score was 4.4 out of 10 during the treatments. Overall, treatments were found to be tolerable, and no subjects had their treatments end early due to pain.

### 3.6 Safety and Adverse Events

Throughout the study period, there were no serious unanticipated adverse events that were reported by any subject. Expected treatment effects were typically observed, including transient erythema, transient edema, and transient burning/stinging sensation. A single case of prolonged hyperpigmentation was reported in a male patient who was FST III and treated in April in Florida, which resolved with subsequent medical intervention that included skin lightening agents. There were no cases of bruising, hair loss, ulceration, necrosis, or scarring.

### 3.7 Fitzpatrick Skin Type Subgroup Analysis

Subgroup analysis demonstrated notable improvements in acne scarring among patients with FST IV–VI, with minimal adverse events reported. A two‐tailed *Z*‐Test (*α* = 0.05) revealed no statistically significant differences between FST I–III and IV–VI across blinded evaluations, ECCA scores, and adverse event rates. When comparing the number of subjects who reported an adverse event, there were no statistically significant differences (*p* > 0.05) found when comparing between lighter and darker skin types. These findings indicate the potential for consistent treatment responses across a broad range of skin tones.

## 4 Discussion

The findings of this study support the utility of this novel 1550 nm non‐ablative resurfacing laser that uses Focal Point Technology to treat acne scars. With acne scars, dermal collagen and elastin disruption and disorganized dermal architecture contribute to its clinical appearance [17]. Therefore, clinical treatments have traditionally focused on methods that can stimulate new collagen and elastin formation. It is generally believed that treatments that can target deeper layers of skin can lead to optimal clinical improvement, especially for atrophic acne scars.

The 1550 nm non‐ablative surfacing laser has long been used to induce dermal coagulation and tissue remodeling to treat fine lines, wrinkles, skin texture, surgical scars, and acne scars. Traditional 1550 nm resurfacing lasers have only been cleared to treat up to 70 mJ. This is due to limits in technology, where traditional laser beams are only oriented vertically in the skin and must pass through the superficial layers to travel deeper down. These superficial layers are limited in their thermal capacity before undergoing detrimental effects, such as burns, necrosis, and scarring. With this new‐generation Focal Point Technology, higher energies, up to 150 mJ, can now safely be delivered to target deeper focal depths. The superficial layers are spared from higher fluences, as the laser beam energy is spatially spread over larger areas above the intended target in a conical configuration.

**FIGURE 2:**
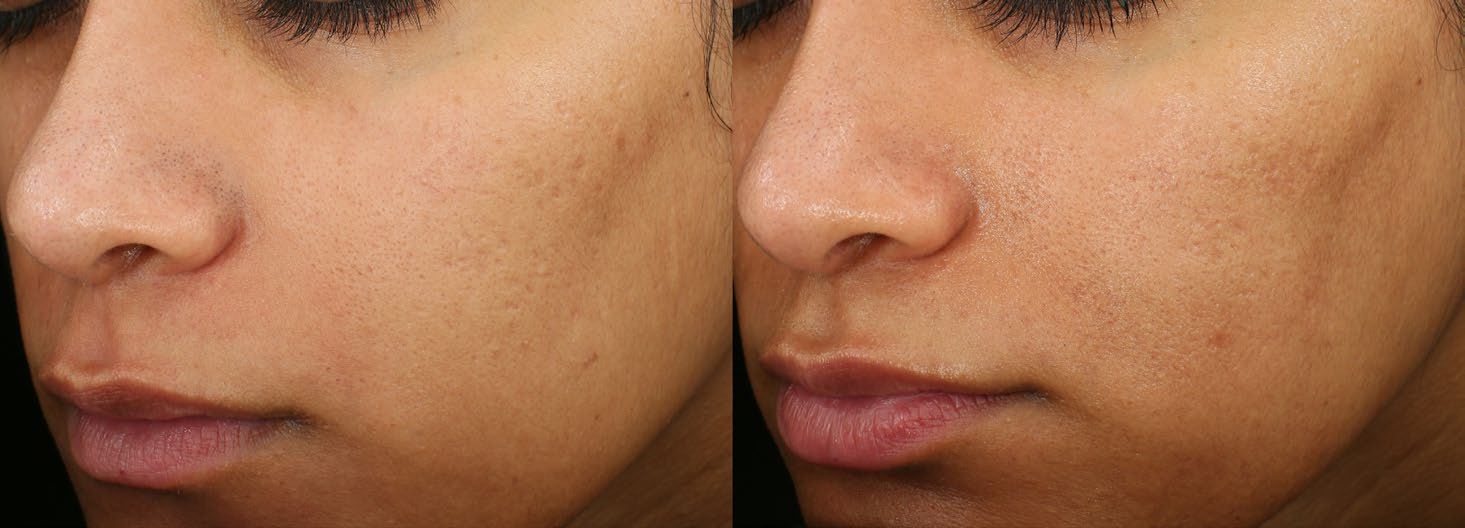
Photographs of 28‐year‐old Asian female, FST IV, at baseline (left) and follow‐up (right) demonstrating 60% reduction in acne scars after 3 treatments.

**FIGURE 3:**
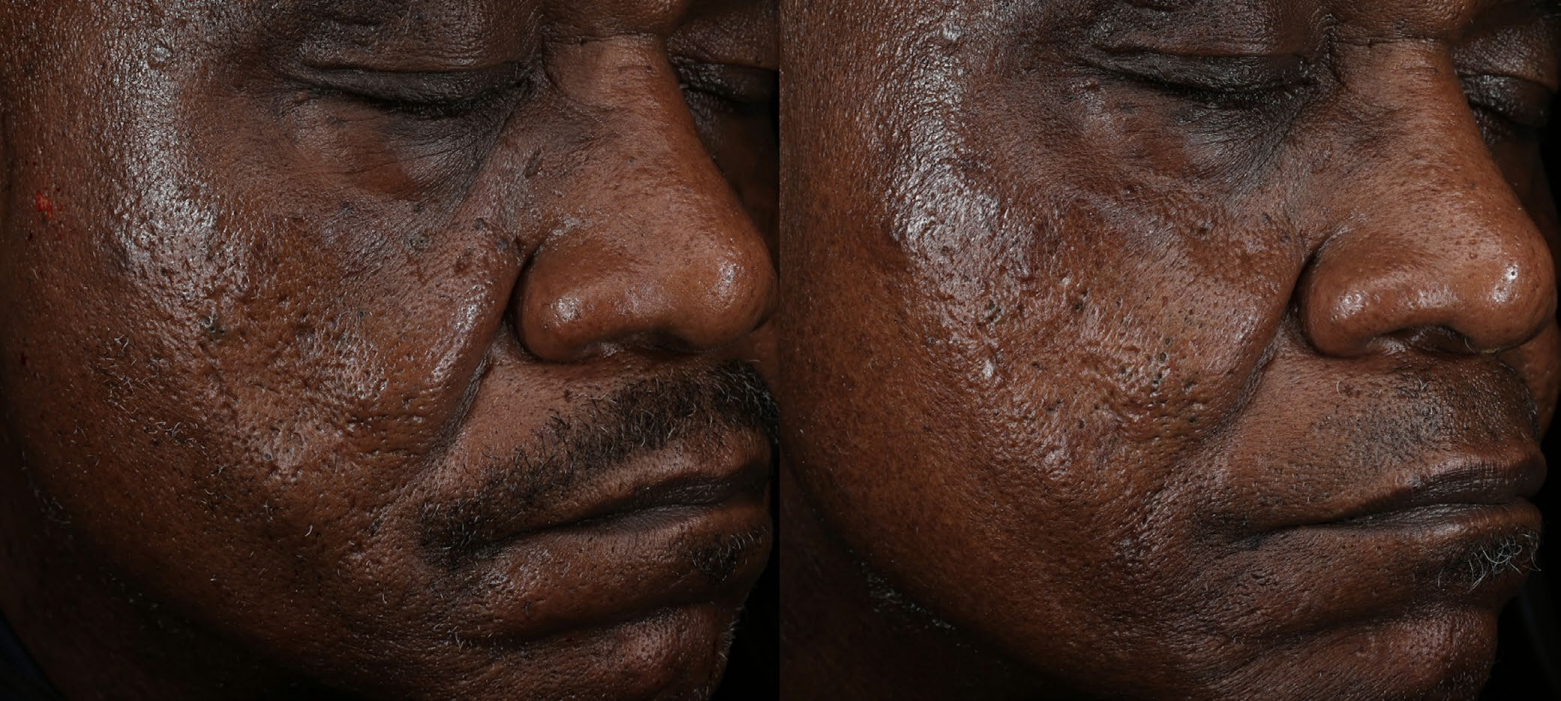
Photographs of 67‐year‐old African American male, FST VI, at baseline (left) and follow‐up (right) demonstrating a 45% reduction in acne scars after 4 treatments.

The subjects included in this study represent FST I–VI, which supports the safe use of this novel 1550 nm non‐ablative resurfacing laser in all skin types (Figure 2 and Figure 3). There were no significant safety concerns raised throughout the duration of the study, and treatments were well tolerated by all subjects. The single case of prolonged hyperpigmentation was witnessed in an FST III male in Florida, who started treatment in the Spring and had subsequent follow‐up in the Summer. The skin type, geographic location, and seasonality may have contributed to this occurrence. Still, the rate of prolonged hyperpigmentation seen in this trial (2.1%) is lower than others reported in the literature for traditional 1550 nm resurfacing lasers (57.1% and 40.0%) [14, 22].

The safety of this novel 1550 nm non‐ablative resurfacing laser in all skin types relies heavily upon both its delivery mechanism and its cooling technology. As previously mentioned, the method of using conical beams for energy delivery helps to limit undesired heat buildup and thermal damage in superficial layers of the skin. Especially when treating skin of color, excess heat buildup can stimulate melanocytes and increase the risk for dyspigmentation, specifically PIH. The uniquely integrated contact cooling includes a sapphire window that is directly incorporated into the handpiece. The surface of this window has the ability to cool up to −10°C, which is essential for adequate protection of the skin surface and subject comfort when delivering high amounts of energy. Excessive thermal damage superficially can contribute to dyspigmentation, blistering, burns, necrosis, and scarring.

The current findings include treatments performed early on when this novel 1550 nm non‐ablative resurfacing laser was still being developed. Since then, the treatment protocols, including laser parameters, treatment techniques, treatment intervals, and peri‐procedural care, have been refined and further optimized. For example, only a single treatment layer was targeted in the early treatments, while current protocols now include treating both a deeper and a superficial layer in the same treatment session. Our clinical outcomes and patient experiences have continued to improve. Future research should further evaluate not just acne scars with these refined protocols, but also other clinical indications, such as fine lines, wrinkles, skin laxity, volumization, surgical scars, and striae. These can include both facial and non‐facial treatment sites and would mimic our real‐world clinic treatments.

## 5 Conclusion

In this multi‐center, prospective trial, treatment with a new‐generation 1550 nm non‐ablative resurfacing laser that uses Focal Point Technology can safely and effectively improve the appearance of acne scars, even when delivering high energies. This study contributes to the growing body of evidence suggesting that advanced laser energy delivery with Focal Point Technology and enhanced cooling may offer consistent acne scar treatment outcomes across all skin types, with particular relevance for populations traditionally more prone to dyspigmentation.

## Author Contributions

All authors have read and approved the final manuscript.

## Funding

This study was supported by AVAVA.

## Ethics Statement

This study was approved by an IRB, and informed consent was obtained from all subjects. Consent for use of photography was gathered from subjects pictured.

## Conflicts of Interest

Jordan V. Wang and Roy G. Geronemus are on the advisory board for AVAVA. Hyemin Pomerantz and Thang‐Nga Tran are consultants for AVAVA. Leyda Bowes is the medical director for AVAVA.

## Data Availability

The data that support the findings of this study are available on request from the corresponding author. The data are not publicly available due to privacy or ethical restrictions.
